# Supplementary material for: Type I interferon drives T cell responses to amyloid beta in the central nervous system
Source: Nat Commun. 2026 Apr 23;17:3737. doi: 10.1038/s41467-026-72262-6 (PMC13106858; doi:10.1038/s41467-026-72262-6)
Supplement: Supplementary file 1 — Supplementary Information [file 41467_2026_72262_MOESM1_ESM.pdf]

Type-I Interferon drives T-cell responses to amyloid-beta in the central nervous system

Supplementary Information

Supplementary Fig. 1: Plaque characterization of APP23 mouse model

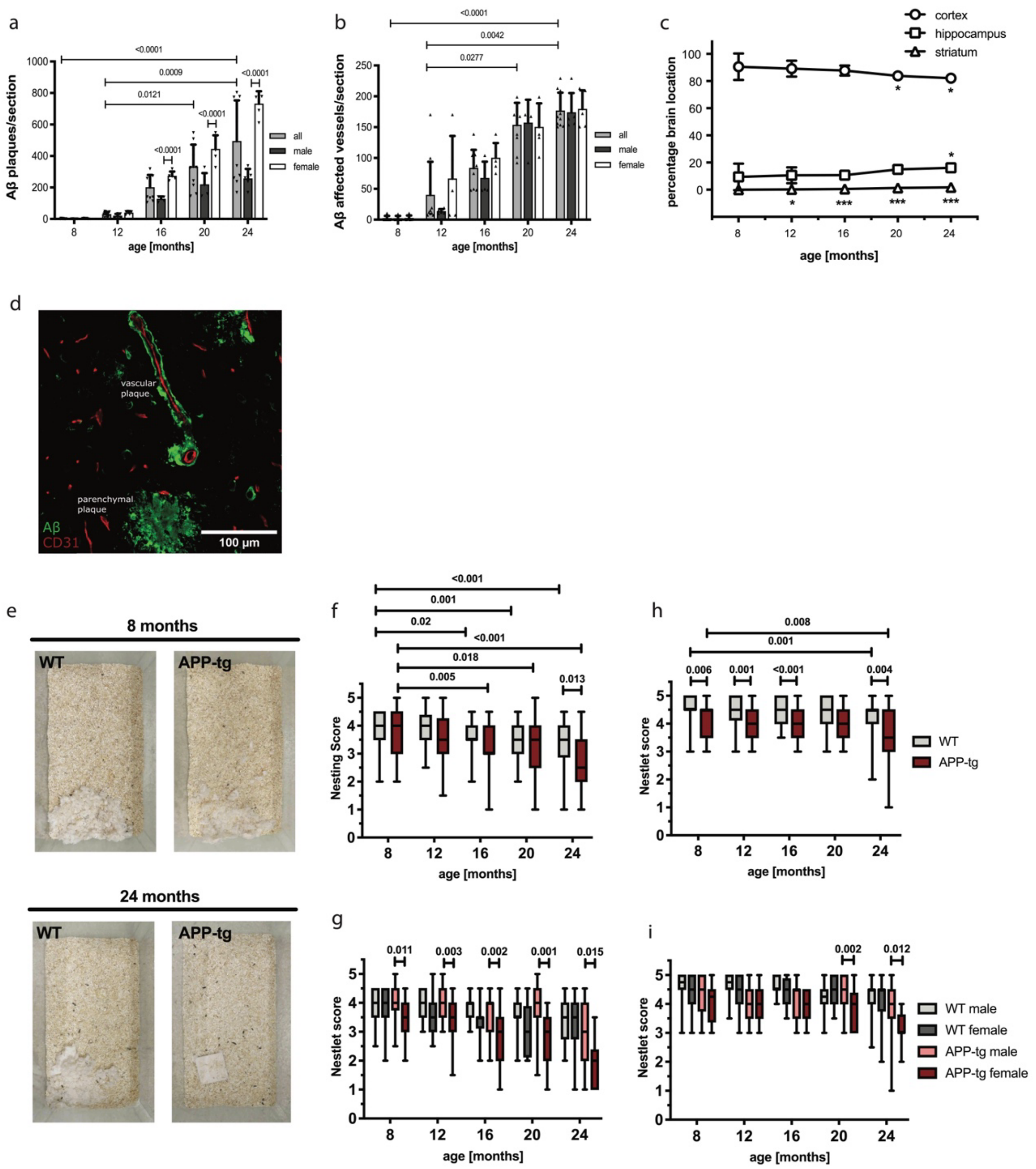

(a) Quantification of Aβ plaques per imaged cortical section across different time points, stratified by sex. A total of n = 10 (n=5 male, n=5 female) sections per time point were analyzed. Statistical significance was assessed using one-way ANOVA and Kruskal–Wallis tests with multiple comparisons. **(b)** Quantification of Aβ-positive blood vessels per section over time, stratified by sex. n = 10 (n=5 male, n=5 female) sections per time point were analyzed. Statistical analysis was performed using one-way ANOVA and Kruskal–Wallis tests with multiple comparisons. **(c)** Overview of the anatomical distribution of Aβ plaque deposition across brain regions as a function of age. **(d)** Representative immunofluorescence (IF) image of a fixed frozen cortical section from a 24-month-old APP23-tg mouse showing parenchymal Aβ plaques (Aβ^{+}) and vascular amyloid deposits (Aβ^{+}CD31^{+}). **(e)** Representative images from

the nestlet shredding test 24 hours after nestlet placement, comparing 8-month-old to 24-month-old APP23-tg mice. **(f)** and **(g)** Quantification of nesting scores 24 hours after nestlet placement. n = 43-47 APP23-tg and n = 40-42 WT mice per time point. **(f)** Comparison between genotypes. **(g)** Additional stratification by sex within each genotype (n=20-25 male, n=20-22 female). **(h)** and **(i)** Quantification of nesting scores 7 days after nestlet placement. n = 43-47 APP23-tg and n = 40-42 WT mice per time point. **(h)** Comparison between genotypes. **(i)** Additional comparison between male and female mice within each genotype (n=20-25 male, n=20-22 female).

Supplementary Fig. 2: Cell type annotation of scRNAseq dataset

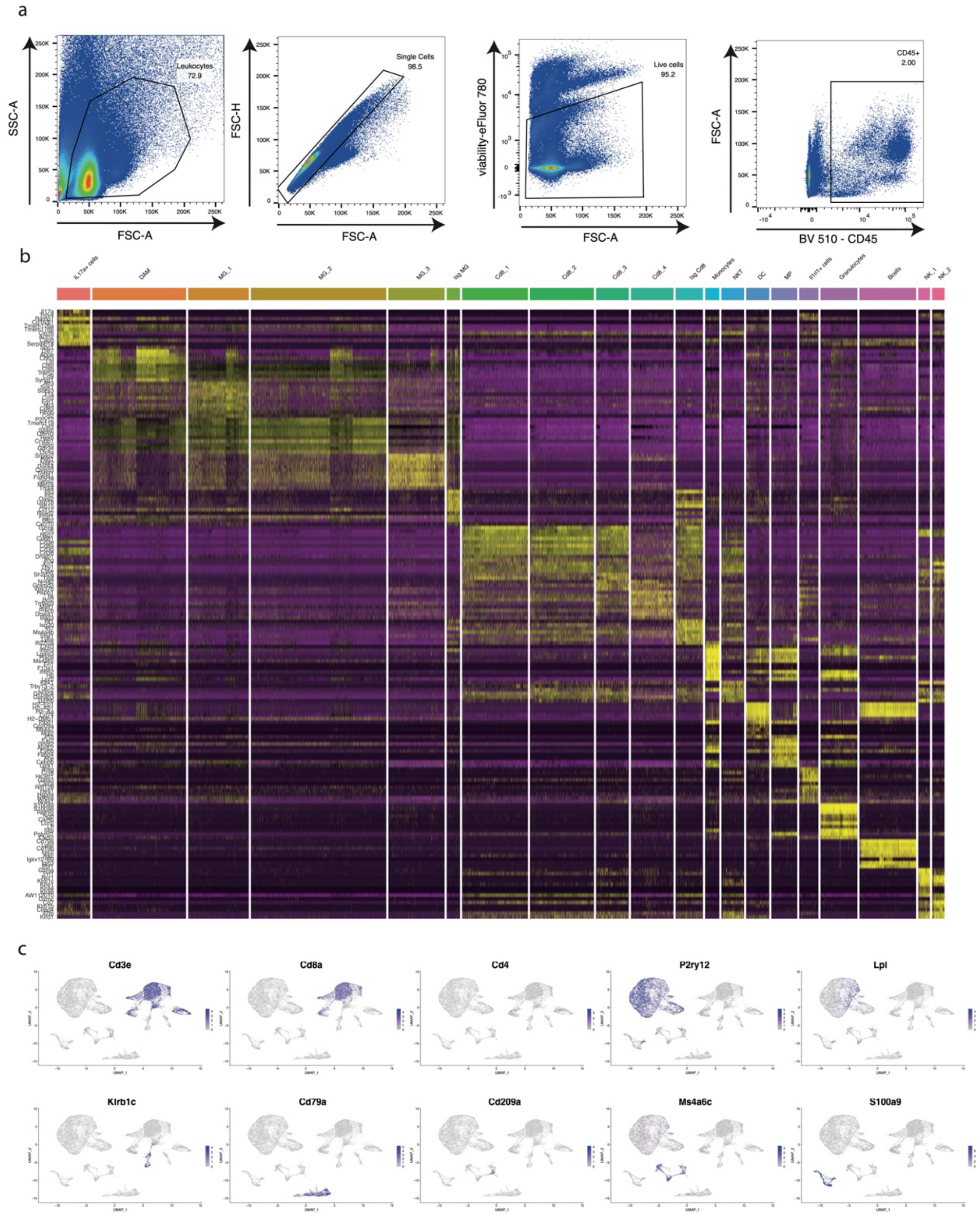

(a) Representative gating strategy used for isolation of CD45^{+} immune cells from brain parenchyma via fluorescence-activated cell sorting (FACS). Sorted cells were processed using the 10x Genomics 5′ single-cell RNA-sequencing workflow. **(b)** Heatmap showing the top 10 differentially expressed genes for each of the 11 transcriptionally defined immune cell clusters identified in the dataset. Yellow indicates upregulated expression within the respective cluster. **(c)** Uniform Manifold Approximation and Projection (UMAP) plots of CD45^{+} immune cells with overlaid expression density gradients for canonical marker genes of T cells, microglia, B cells, dendritic cells, NK cells, and monocytes/macrophages, illustrating the transcriptional identity of each cell population.

Supplementary Fig. 3: Frequencies of cell types in APP23-tg and wild-type mice

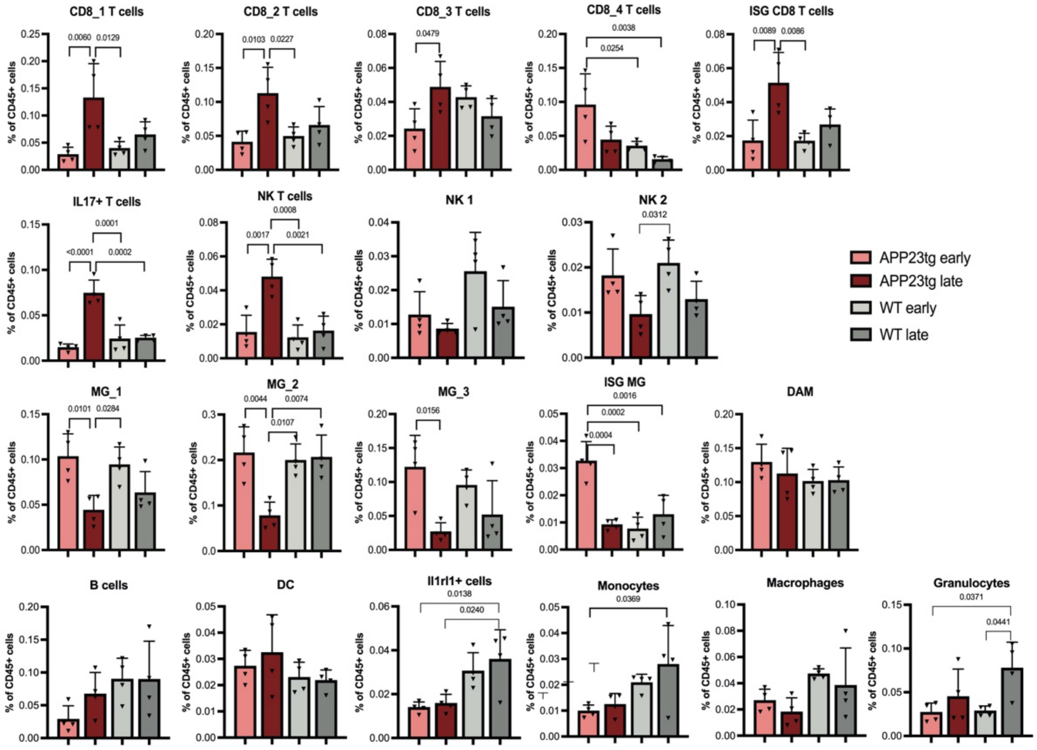

Bar plots showing the frequency of each identified immune cell subset within the total CD45^{+} population across all cohorts. Individual data points represent values from each mouse (n = 4 per cohort). Statistical significance was assessed using ordinary two-way ANOVA followed by Tukey's multiple comparisons test. P-values are indicated on the plots.

Supplementary Fig. 4: IL17+ T-cells in APP23 mouse model

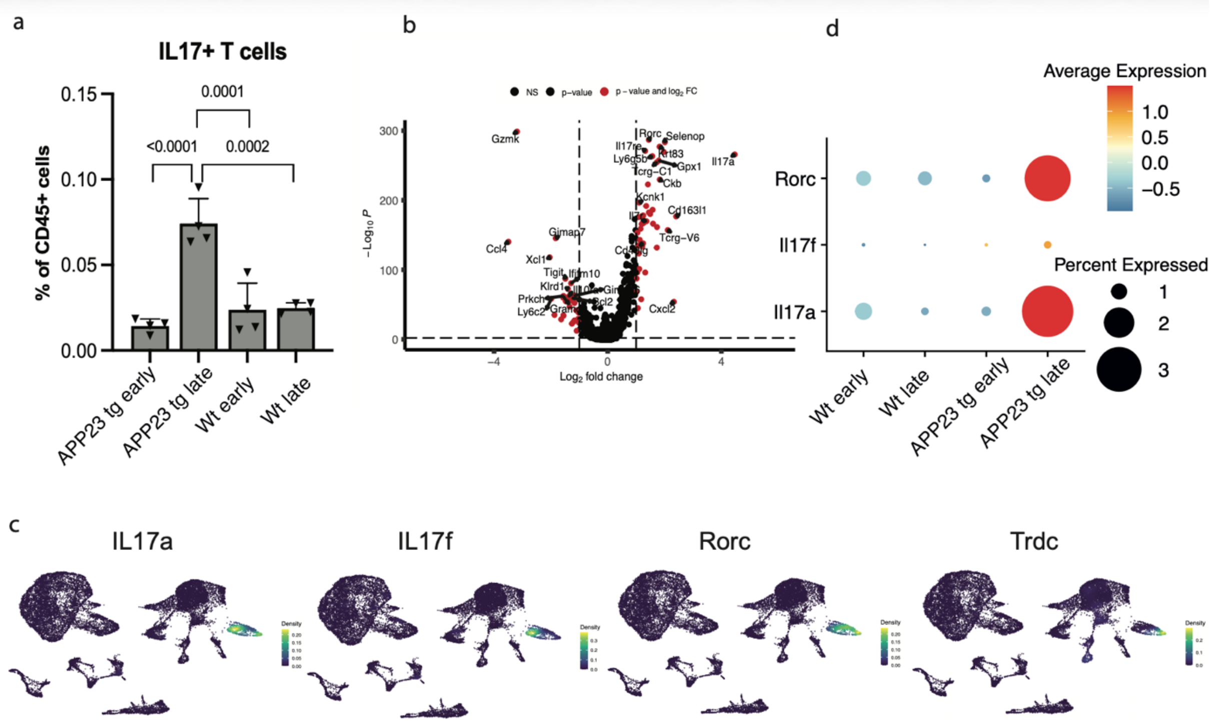

(a) Bar plot showing the frequency of IL-17⁺ cells within the CD45⁺ immune cell population across four experimental groups: APP23-tg early, APP23-tg late, wild-type (WT) early, and WT late. n = 4 mice per group. Statistical significance was assessed using ordinary two-way ANOVA with Tukey's multiple comparisons test; p-values are indicated. **(b)** Volcano plot displaying differentially expressed genes between IL-17⁺ cells and all other immune cell subsets. Upregulated and downregulated genes are highlighted based on fold change and adjusted p-values. **(c)** Uniform Manifold Approximation and Projection (UMAP) plots of CD45+ immune cells. The density gradient overlay highlights the average expression of the following genes: IL17a, IL17f, Rorc and Trdc. **(d)** Dot plot showing average RNA expression (color scale) and expression frequency (dot size) of Rorc, Il17a, and Il17f across all cohorts.

Supplementary Fig. 5: Immunohistological assessment of T-cell infiltration

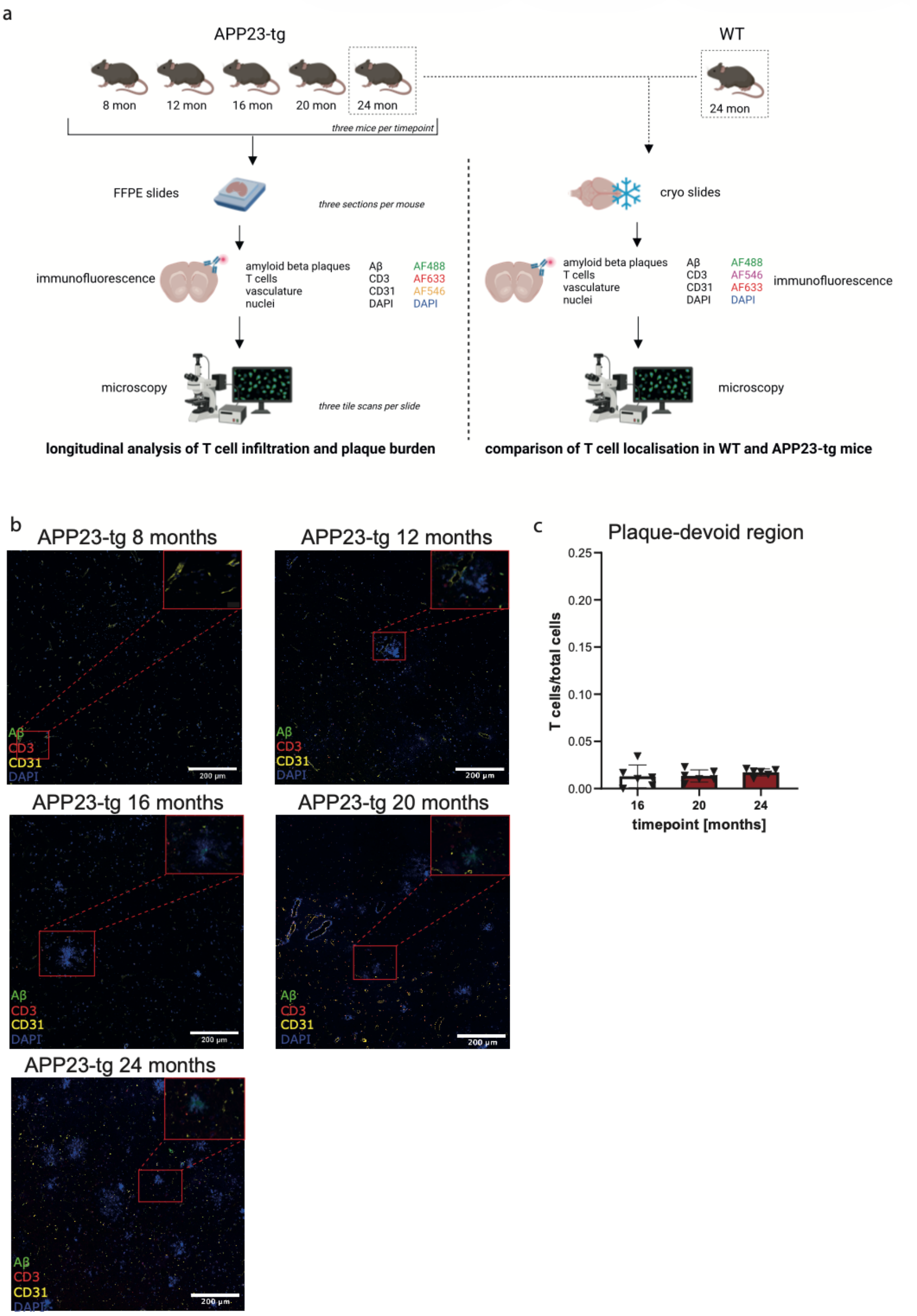

(a) Experimental setup of immunohistological analysis of T cell infiltration and plaque burden. Wt and APP23-tg mice of different ages were sacrificed to obtain either FFPE or cryopreserved brain tissue sections. These were stained as indicated in the methods section and were analyzed on an LSM700 confocal microscope. **(b)** Representative tile scans (960.25 µm²) and corresponding zoomed-in regions of interest from FFPE cortical sections of APP23-tg mice aged 8, 12, 16, 20, and 24 months. All images were acquired at 20× magnification using an LSM700 confocal microscope. Zoomed-in areas are outlined in red. **(c)** Quantification of $CD3^{+}$ T cells located in plaque-devoid brain regions at 16, 20, and 24 months. T-cell counts were normalized to total cell numbers per field based on CD3 staining.

Supplementary Fig. 6: Spatial transcriptomic analysis and characterization of ISG$^+$ T cells in APP23-tg mouse brain

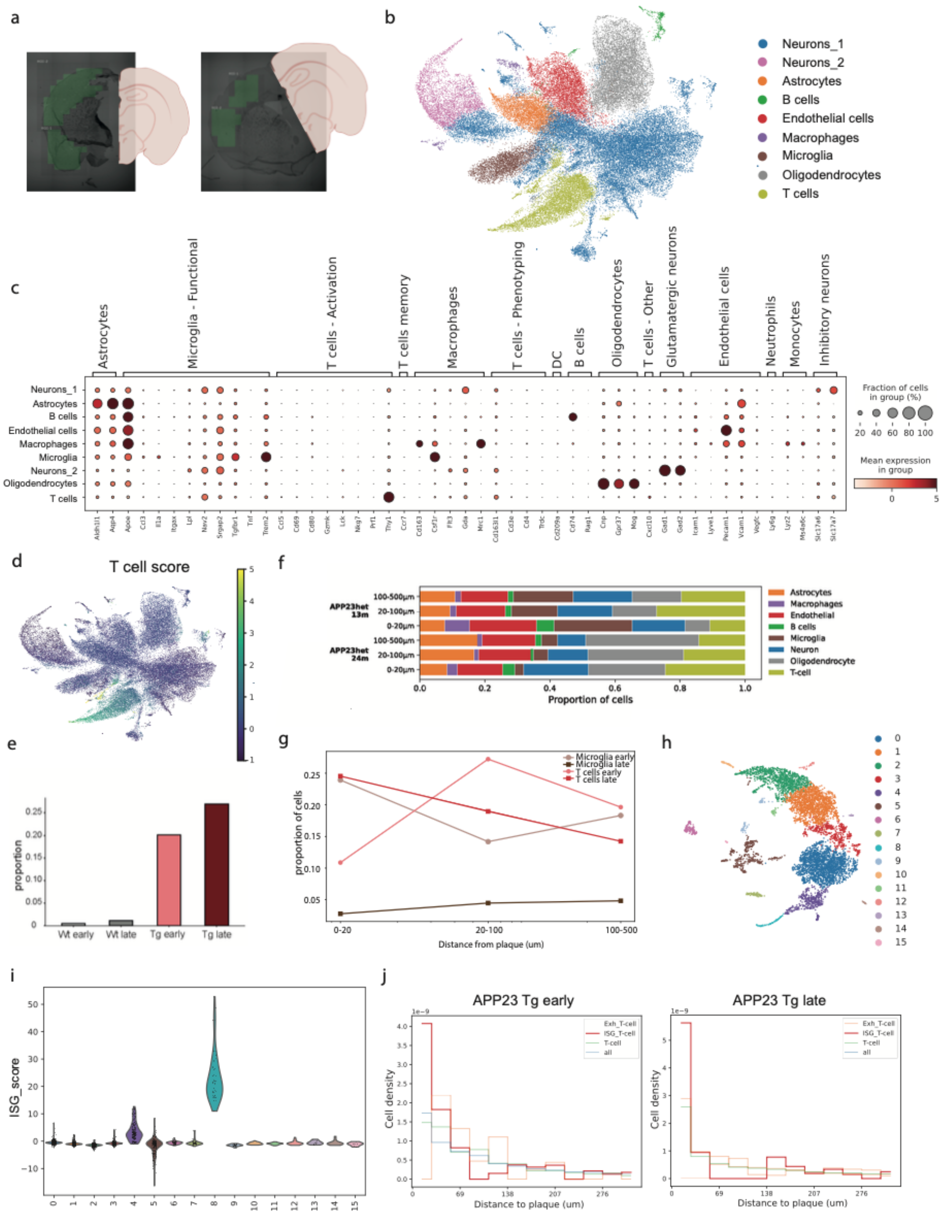

(a) Schematic illustration of regions of interest (ROIs) selected for targeted spatial transcriptomic profiling on two representative APP23-tg mouse brain sections. (**b**) Uniform Manifold Approximation and Projection (UMAP) plot showing all integrated cells obtained from spatial RNA sequencing colored by Leiden cluster identity. **(c)** Dot plot highlighting the expression of canonical cell type marker genes across identified clusters. Dot size represents expression frequency; color represents mean average expression per group. **(d)** Uniform Manifold Approximation and Projection (UMAP) plot of all integrated cells obtained from spatial RNA sequencing with overlaid expression density gradient for canonical T cell marker. **(e)** Bar plot showing proportion of T cell cluster across experimental groups within all integrated cells obtained from spatial RNA sequencing **(f)** Stacked bar plots showing the proportional composition of major cell types identified by spatial transcriptomics stratified by distance from amyloid plaques (0-20 μm, 20-100 μm, and 100-500 μm) in APP23het mice at 13 months (n=2) and 24 months (n=2) of age. **(g)** Line plots illustrating the proportion of microglia and T cells across increasing distances from amyloid plaques (0-20 μm, 20-100 μm, and 100-500 μm) in APP23het mice at 13 months (early, n=2) and 24 months (late, n=2). **(h)** Uniform Manifold

Approximation and Projection (UMAP) visualization of T cell cluster from **(b)** colored by Leiden cluster identity. **(i)** Violin plot showing ISG_score expression levels across all Lieden clusters identified in **(h)**. **(j)** Cell density distribution relative to plaque edge, comparing all cells, ISG^{+} T cells, exhausted T cells and other T cells in APP23tg mice.

Supplementary Fig. 7: Spatial transcriptomic analysis and characterization of human Alzheimer's samples

a

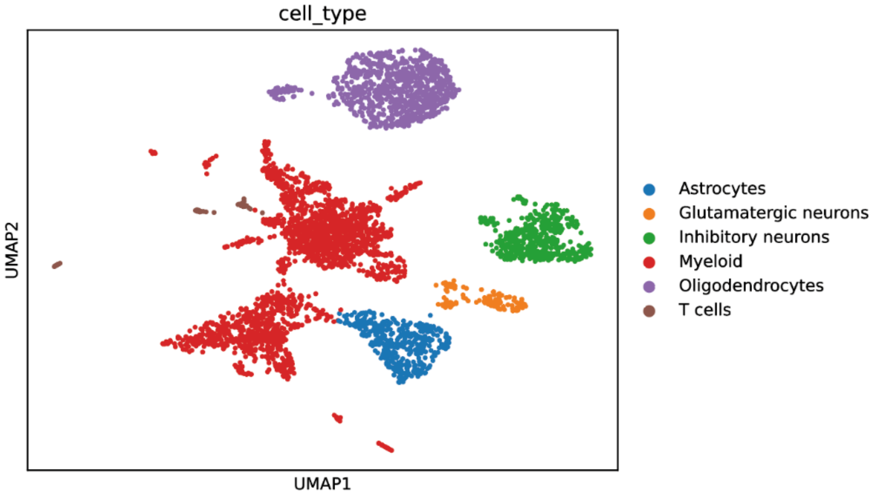

b

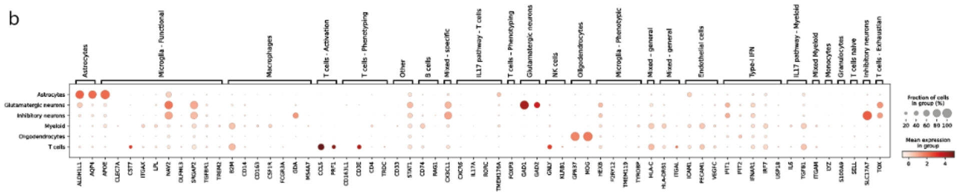

(**a**) Uniform Manifold Approximation and Projection (UMAP) plot of all integrated cells obtained from spatial RNA sequencing of cortical lobe tissue from human patients (n = 4), colored by Leiden cluster identity. **(b)** Dot plot highlighting the expression of canonical cell type marker genes across identified clusters. Dot size represents expression frequency; color represents mean average expression per group.

Supplementary Fig. 8: Clonal distribution of top expanded T-cell clones in late-stage APP23-tg mice.

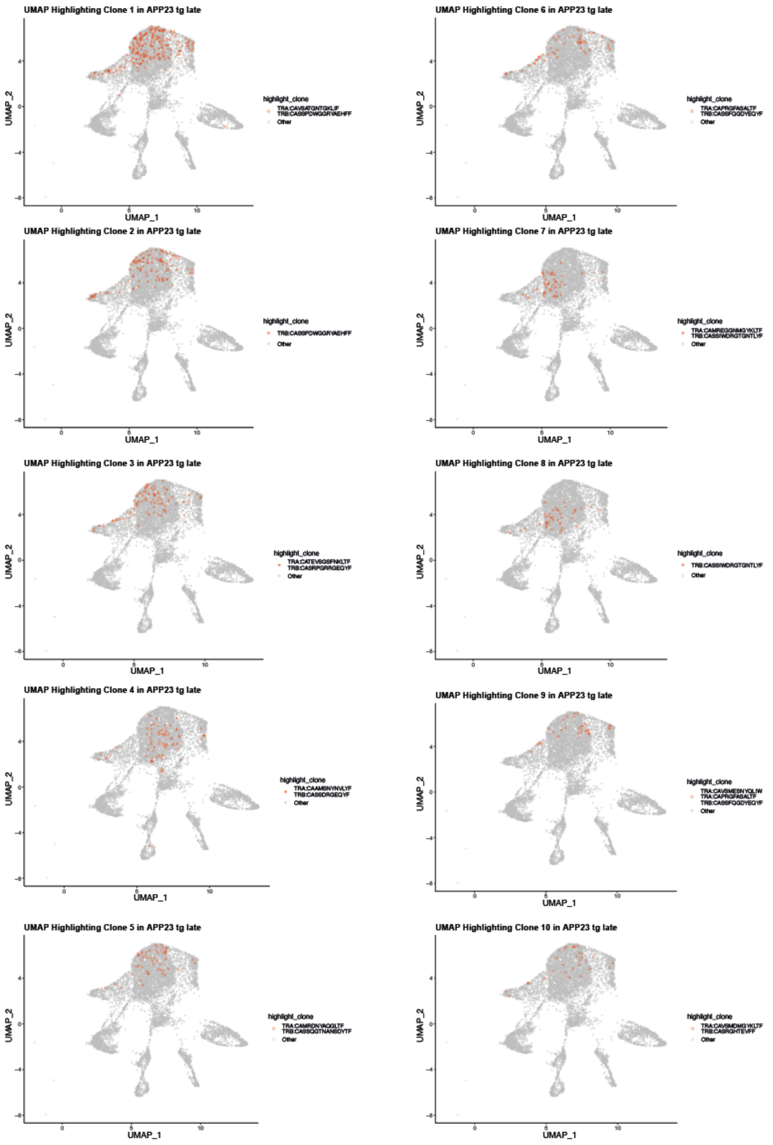

UMAP plots showing the spatial distribution of the top 10 most expanded T-cell clones in the APP23-tg late cohort, overlaid onto transcriptionally defined clusters. Each plot highlights the localization of a single dominant clone, illustrating clonal enrichment across distinct T-cell subsets.

Supplementary Fig. 9: CRISPR-based CXCR3 knock-out in murine T-cells and transwell assays

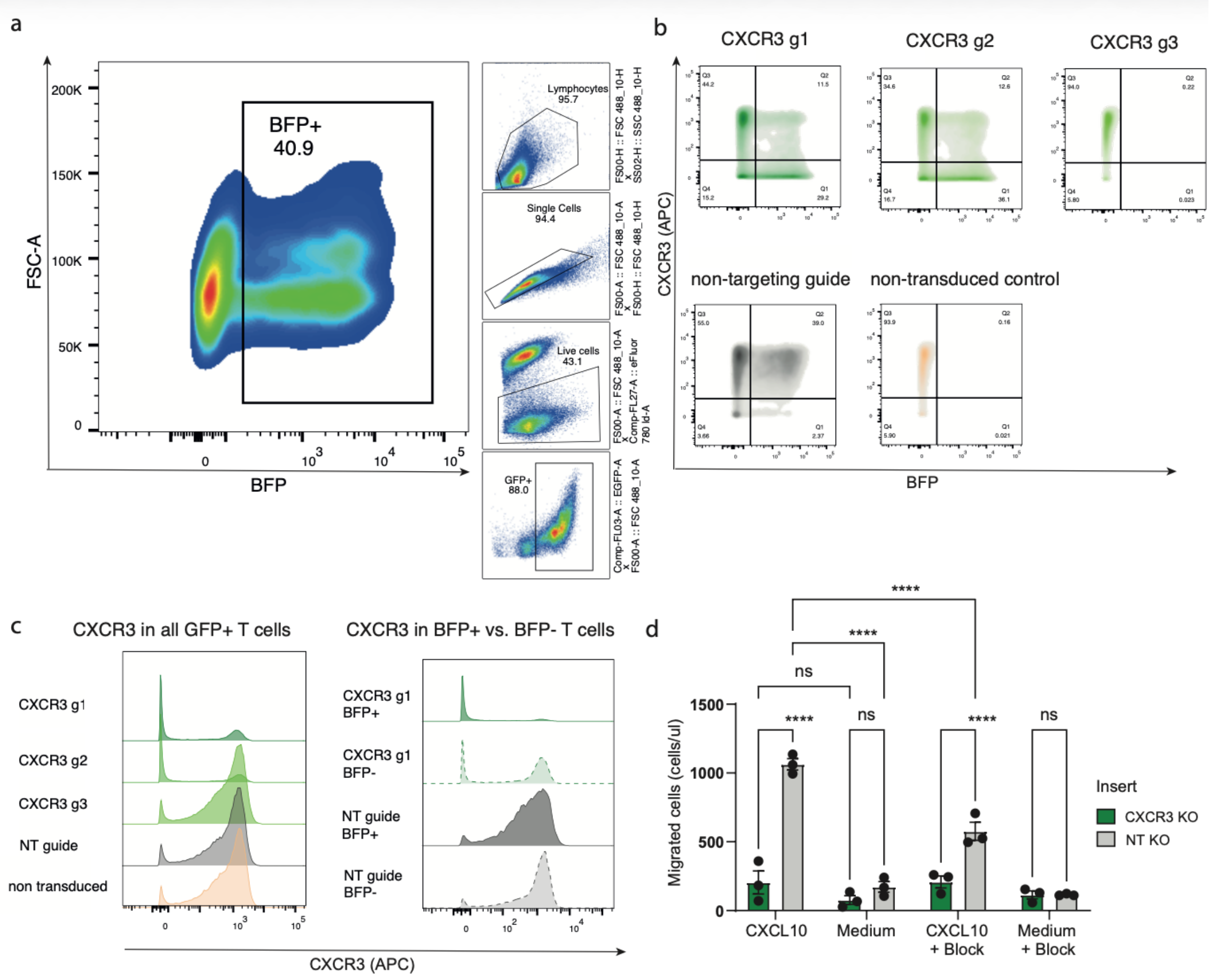

(a) Representative flow cytometry gating strategy for identifying BFP⁺ transduced T cells. Sequential gating was applied for lymphocytes, singlets, live cells, GFP⁺ and BFP⁺ populations. The box highlights the percentage of BFP⁺ cells, indicating successful transduction. **(b)** Flow cytometric analysis of CXCR3 surface expression in T cells transduced with three different CXCR3-targeting gRNAs (g1–g3), a non-targeting (NT) control guide, or non-transduced. CXCR3 (APC, y-axis) versus eBFP (x-axis) staining shows reduced CXCR3 signal in CXCR3 gRNA-transduced cells compared to controls. **(c)** Histograms showing CXCR3 expression in GFP⁺ T cells transduced with CXCR3 gRNAs (green), NT guide (gray), or non-transduced cells (orange). Left: CXCR3 expression in all GFP⁺ T cells. Right: comparison of CXCR3 levels between BFP⁺ (successfully transduced) and BFP⁻ (non-transduced) subsets for CXCR3 g1 and NT guide conditions, confirming efficient knockout in BFP⁺ cells. **(d)** Quantification of migrated T cells in response to CXCL10 in a transwell assay. CXCR3 knockout (green bars) significantly reduced migration toward CXCL10 compared to NT controls (gray bars). Migration was abrogated by CXCR3 blocking antibody ("Block") and not observed in medium-only controls. Data represent mean ± SD; statistical significance was assessed by two-way ANOVA with Tukey's multiple comparisons test, ****$p < 0.0001$, ns = not significant.

Supplementary Table 1: List of genes probed for murine targeted spatial transcriptomics

#	Species	Gene	Gene ID
1	Mm	Cd3e	ENSMUSG00000032093
2	Mm	Cd4	ENSMUSG00000023274
3	Mm	Cd8b1	ENSMUSG00000053044
4	Mm	Foxp3	ENSMUSG00000039521
5	Mm	Il17a	ENSMUSG00000025929
6	Mm	Il17f	ENSMUSG00000041872
7	Mm	Rorc	ENSMUSG00000028150
8	Mm	Trdc	ENSMUSG00000104876
9	Mm	Cd163l1	ENSMUSG00000025461
10	Mm	Isg15	ENSMUSG00000035692
11	Mm	Ifit3	ENSMUSG00000074896
12	Mm	Cst3	ENSMUSG00000027447
13	Mm	Hexb	ENSMUSG00000021665
14	Mm	Tyrobp	ENSMUSG00000030579
15	Mm	Ctsd	ENSMUSG00000007891
16	Mm	P2ry12	ENSMUSG00000036353
17	Mm	Tmem119	ENSMUSG00000054675
18	Mm	Tgfbr1	ENSMUSG00000007613
19	Mm	Olfml3	ENSMUSG00000027848
20	Mm	Cst7	ENSMUSG00000068129
21	Mm	Apoe	ENSMUSG00000002985
22	Mm	Lpl	ENSMUSG00000015568
23	Mm	Trem2	ENSMUSG00000023992
24	Mm	Srgap2	ENSMUSG00000026425
25	Mm	Nav2	ENSMUSG00000052512
26	Mm	Ccl3	ENSMUSG00000000982
27	Mm	Il1a	ENSMUSG00000027399
28	Mm	Tnf	ENSMUSG00000024401
29	Mm	Ifit2	ENSMUSG00000045932
30	Mm	Irf7	ENSMUSG00000025498
31	Mm	Cxcl10	ENSMUSG00000034855
32	Mm	Ifnb1	ENSMUSG00000048806
33	Mm	Ifnar1	ENSMUSG00000022967

34	Mm	Cd79a	ENSMUSG00000003379
35	Mm	Rag1	ENSMUSG00000061311
36	Mm	Cd74	ENSMUSG00000024610
37	Mm	H2-Ab1	ENSMUSG00000073421
38	Mm	H2-K1	ENSMUSG00000061232
39	Mm	H2-D1	ENSMUSG00000073411
40	Mm	Mrc1	ENSMUSG00000026712
41	Mm	Gda	ENSMUSG00000058624
42	Mm	Csf1r	ENSMUSG00000024621
43	Mm	Flt3	ENSMUSG00000042817
44	Mm	Ms4a7	ENSMUSG00000024672
45	Mm	Cd163	ENSMUSG00000008845
46	Mm	Lyz2	ENSMUSG00000069516
47	Mm	Ms4a6c	ENSMUSG00000079419
48	Mm	Itgax	ENSMUSG00000030789
49	Mm	Cd209a	ENSMUSG00000031494
50	Mm	Icam1	ENSMUSG00000037405
51	Mm	Pecam1	ENSMUSG00000020717
52	Mm	Lyve1	ENSMUSG00000030787
53	Mm	Vegfc	ENSMUSG00000031520
54	Mm	Vcam1	ENSMUSG00000027962
55	Mm	S100a9	ENSMUSG00000056071
56	Mm	Ly6g	ENSMUSG00000022582
57	Mm	Ncr1	ENSMUSG00000062524
58	Mm	Klrb1c	ENSMUSG00000030325
59	Mm	Gzma	ENSMUSG00000023132
60	Mm	Gzmk	ENSMUSG00000042385
61	Mm	Nkg7	ENSMUSG00000004612
62	Mm	Ccl5	ENSMUSG00000035042
63	Mm	Ifng	ENSMUSG00000055170
64	Mm	Thy1	ENSMUSG00000032011
65	Mm	Tigit	ENSMUSG00000071552
66	Mm	Tox	ENSMUSG00000041272
67	Mm	Pdcd1	ENSMUSG00000026285
68	Mm	Prf1	ENSMUSG00000037202
69	Mm	Lck	ENSMUSG00000000409
70	Mm	Cd69	ENSMUSG00000030156

71	Mm	Cd40lg	ENSMUSG00000031132
72	Mm	Tmem176a	ENSMUSG00000023367
73	Mm	Cxcr6	ENSMUSG00000048521
74	Mm	Il7r	ENSMUSG00000003882
75	Mm	Mki67	ENSMUSG00000031004
76	Mm	Il6	ENSMUSG00000025746
77	Mm	Tgfb1	ENSMUSG00000002603
78	Mm	Itgam	ENSMUSG00000030786
79	Mm	Itgal	ENSMUSG00000030830
80	Mm	Ccr7	ENSMUSG00000037944
81	Mm	Cx3cr1	ENSMUSG00000052336
82	Mm	Cx3cl1	ENSMUSG00000031778
83	Mm	Mmp9	ENSMUSG00000017737
84	Mm	Cd33	ENSMUSG00000004609
85	Mm	Cd80	ENSMUSG00000075122
86	Mm	Cd86	ENSMUSG00000022901
87	Mm	Sell	ENSMUSG00000026581
88	Mm	Il1b	ENSMUSG00000027398
89	Mm	Cd68	ENSMUSG00000018774
90	Mm	Stat1	ENSMUSG00000026104
91	Mm	Aldh1l1	ENSMUSG00000030088
92	Mm	Aqp4	ENSMUSG00000024411
93	Mm	Mog	ENSMUSG00000076439
94	Mm	Gpr37	ENSMUSG00000039904
95	Mm	Cnp	ENSMUSG00000006782
96	Mm	Gad2	ENSMUSG00000026787
97	Mm	Gad1	ENSMUSG00000070880
98	Mm	Slc17a7	ENSMUSG00000070570
99	Mm	Slc17a6	ENSMUSG00000030500
100	Mm	Trbc1	ENSMUSG00000076490

Supplementary Table 2: List of genes probed for human targeted spatial transcriptomics

#	Species	Gene	Gene ID
1	Hs	CD3E	ENSG00000198851
2	Hs	CD4	ENSG00000010610
3	Hs	CD8A	ENSG00000153563
4	Hs	FOXP3	ENSG00000049768
5	Hs	TRDC	ENSG00000211829
6	Hs	CD163L1	ENSG00000177675
7	Hs	CCR7	ENSG00000126353
8	Hs	SELL	ENSG00000188404
9	Hs	CD74	ENSG00000019582
10	Hs	RAG1	ENSG00000166349
11	Hs	NCR1	ENSG00000189430
12	Hs	KLRB1	ENSG00000111796
13	Hs	IL17A	ENSG00000112115
14	Hs	IL17F	ENSG00000112116
15	Hs	RORC	ENSG00000143365
16	Hs	TMEM176A	ENSG00000002933
17	Hs	CXCR6	ENSG00000172215
18	Hs	IL7R	ENSG00000168685
19	Hs	IL6	ENSG00000136244
20	Hs	TGFB1	ENSG00000105329
21	Hs	GZMB	ENSG00000100453
22	Hs	GZMK	ENSG00000113088
23	Hs	NKG7	ENSG00000105374
24	Hs	IFNG	ENSG00000111537
25	Hs	THY1	ENSG00000154096
26	Hs	TIGIT	ENSG00000181847
27	Hs	TOX	ENSG00000198846
28	Hs	PDCD1	ENSG00000188389
29	Hs	PRF1	ENSG00000180644
30	Hs	LCK	ENSG00000182866
31	Hs	CD69	ENSG00000110848
32	Hs	CD40LG	ENSG00000102245

33	Hs	CD80	ENSG00000121594
34	Hs	CXCL10	ENSG00000169245
35	Hs	IFIT1	ENSG00000185745
36	Hs	IFIT2	ENSG00000119922
37	Hs	IRF7	ENSG00000185507
38	Hs	IFNB1	ENSG00000171855
39	Hs	IFNAR1	ENSG00000142166
40	Hs	USP18	ENSG00000184979
41	Hs	HEXB	ENSG00000049860
42	Hs	TYROBP	ENSG00000011600
43	Hs	P2RY12	ENSG00000169313
44	Hs	TMEM119	ENSG00000183160
45	Hs	TGFBR1	ENSG00000106799
46	Hs	OLFML3	ENSG00000116774
47	Hs	CST7	ENSG00000077984
48	Hs	LPL	ENSG00000175445
49	Hs	TREM2	ENSG00000095970
50	Hs	SRGAP2	ENSG00000266028
51	Hs	NAV2	ENSG00000166833
52	Hs	IL1A	ENSG00000115008
53	Hs	TNF	ENSG00000232810
54	Hs	ITGAX	ENSG00000140678
55	Hs	MRC1	ENSG00000260314
56	Hs	GDA	ENSG00000119125
57	Hs	CSF1R	ENSG00000182578
58	Hs	FLT3	ENSG00000122025
59	Hs	MS4A7	ENSG00000166927
60	Hs	CD163	ENSG00000177575
61	Hs	ADGRE1	ENSG00000174837
62	Hs	S100A9	ENSG00000163220
63	Hs	ICAM1	ENSG00000090339
64	Hs	PECAM1	ENSG00000261371
65	Hs	LYVE1	ENSG00000133800
66	Hs	VEGFC	ENSG00000150630
67	Hs	VCAM1	ENSG00000162692
68	Hs	ITGAM	ENSG00000169896
69	Hs	ITGAL	ENSG00000005844

70	Hs	CX3CR1	ENSG00000168329
71	Hs	CX3CL1	ENSG00000006210
72	Hs	MKI67	ENSG00000148773
73	Hs	MMP9	ENSG00000100985
74	Hs	CD33	ENSG00000105383
75	Hs	CD86	ENSG00000114013
76	Hs	IL1B	ENSG00000125538
77	Hs	STAT1	ENSG00000115415
78	Hs	HLA-A	ENSG00000206503
79	Hs	HLA-DRB1	ENSG00000196126
80	Hs	ALDH1L1	ENSG00000144908
81	Hs	AQP4	ENSG00000171885
82	Hs	MOG	ENSG00000204655
83	Hs	GPR37	ENSG00000170775
84	Hs	GAD2	ENSG00000136750
85	Hs	GAD1	ENSG00000128683
86	Hs	SLC17A7	ENSG00000104888
87	Hs	SLC17A6	ENSG00000091664
88	Hs	CD209	ENSG00000090659
89	Hs	CD79A	ENSG00000105369
90	Hs	LYZ	ENSG00000090382
91	Hs	CLEC7A	ENSG00000172243
92	Hs	CD14	ENSG00000170458
93	Hs	FCGR3A	ENSG00000203747
94	Hs	APOE	ENSG00000130203
95	Hs	B2M	ENSG00000166710
96	Hs	HLA-C	ENSG00000204525
97	Hs	GNLY	ENSG00000115523
98	Hs	CD27	ENSG00000139193
99	Hs	CCL5	ENSG00000271503
100	Hs	TRBC1	ENSG00000211751

Supplementary Table 3: Clinical information of human AD samples

Patient	Tissue format	Tissue type	Age	Sex	Braak-stage	CERAD	APOE-genotype	CAA Stage according to Thal
756	Cryo-conserved	Cortical region, occipital lobe	70	m	6	C	apoE3/3	2
791	Cryo-conserved	Cortical region, occipital lobe	79	m	4	C	apoE3/3	3
795	Cryo-conserved	Cortical region, occipital lobe	77	m	5	C	apoE3/3	2
825	Cryo-conserved	Cortical region, occipital lobe	76	m	5	C	apoE3/3	2

Supplementary Table 4: sgRNA sequences

sgRNA	Sequence
NT guide	GGAGCTGAGGAGCTGCATAG
Cxcr3_g1	TAGCACCACCAGGTGATAGG
Cxcr3_g2	AGGGCTACACGTACCCGG
Cxcr3_g3	GAGACCCCATACAACTATGC
