# Supplementary material for: Type I interferon drives T cell responses to amyloid beta in the central nervous system
Source: Nat Commun. 2026 Apr 23;17:3737. doi: 10.1038/s41467-026-72262-6 (PMC13106858; doi:10.1038/s41467-026-72262-6)
Supplement: Supplementary file 2 — Reporting Summary [file 41467_2026_72262_MOESM2_ESM.pdf]

nature portfolio

Corresponding author(s): Lukas Bunse

Last updated by author(s): Feb 15, 2026

Reporting Summary

Nature Portfolio wishes to improve the reproducibility of the work that we publish. This form provides structure for consistency and transparency in reporting. For further information on Nature Portfolio policies, see our Editorial Policies and the Editorial Policy Checklist.

Statistics

For all statistical analyses, confirm that the following items are present in the figure legend, table legend, main text, or Methods section.

n/a	Confirmed	
[ ]	[x]	The exact sample size (*n*) for each experimental group/condition, given as a discrete number and unit of measurement
[ ]	[x]	A statement on whether measurements were taken from distinct samples or whether the same sample was measured repeatedly
[ ]	[x]	The statistical test(s) used AND whether they are one- or two-sided<br>*Only common tests should be described solely by name; describe more complex techniques in the Methods section.*
[x]	[ ]	A description of all covariates tested
[ ]	[x]	A description of any assumptions or corrections, such as tests of normality and adjustment for multiple comparisons
[ ]	[x]	A full description of the statistical parameters including central tendency (e.g. means) or other basic estimates (e.g. regression coefficient) AND variation (e.g. standard deviation) or associated estimates of uncertainty (e.g. confidence intervals)
[ ]	[x]	For null hypothesis testing, the test statistic (e.g. *F*, *t*, *r*) with confidence intervals, effect sizes, degrees of freedom and *P* value noted<br>*Give P values as exact values whenever suitable.*
[x]	[ ]	For Bayesian analysis, information on the choice of priors and Markov chain Monte Carlo settings
[x]	[ ]	For hierarchical and complex designs, identification of the appropriate level for tests and full reporting of outcomes
[ ]	[x]	Estimates of effect sizes (e.g. Cohen's *d*, Pearson's *r*), indicating how they were calculated

Our web collection on statistics for biologists contains articles on many of the points above.

Software and code

Policy information about availability of computer code

Data collection	Analysis software and code used from publicly available software is described in the manuscript and listed below. Custom code for downstream spatial transcriptomic analysis is available on request. The methodology has been described in detail in the Methods section of the manuscript.

Data analysis	CellRanger v5.0<br>Seurat v.4.4.0<br>SingleR v2.4.1<br>MAST v1.26.0<br>ggplot2 v3.5.1<br>Celldex v1.10.0<br>CellChat v1.6.1<br>Scanpy v1.10.1<br>Scirpy v0.17.0<br>Tifffile<br>Scipy v1.13.1<br>harmony v0.0.10<br>QIAGEN Ingenuity Pathway Analysis (IPA), accessed 2024<br><br>All parameters and detailed use of packages/software is included in the manuscript.

For manuscripts utilizing custom algorithms or software that are central to the research but not yet described in published literature, software must be made available to editors and reviewers. We strongly encourage code deposition in a community repository (e.g. GitHub). See the Nature Portfolio guidelines for submitting code & software for further information.

Data

Policy information about availability of data

All manuscripts must include a data availability statement. This statement should provide the following information, where applicable:

- Accession codes, unique identifiers, or web links for publicly available datasets
- A description of any restrictions on data availability
- For clinical datasets or third party data, please ensure that the statement adheres to our policy

All single-cell sequencing reads, count matrices and post-processed files are available at the Gene Expression Omnibus (Accession number: GSE280018). All spatial transcriptomic raw data and cell segmented data is available at Zenodo: https://doi.org/10.5281/zenodo.18648333.

Research involving human participants, their data, or biological material

Policy information about studies with human participants or human data. See also policy information about sex, gender (identity/presentation), and sexual orientation and race, ethnicity and racism.

Reporting on sex and gender	Human post-mortem Alzheimer's disease samples used for spatial transcriptomic analyses were all male, reflecting tissue availability through the brain bank at the time of acquisition. Sex was recorded based on donor information provided by the brain bank (as documented in medical records). The study was not designed or powered to assess sex- or gender-specific differences in the human cohort, and no sex-stratified analyses were performed.
Reporting on race, ethnicity, or other socially relevant groupings	N/A
Population characteristics	Provided in the Methods section of the manuscript
Recruitment	Cryopreserved autopsy tissue was used. No active recruitment.
Ethics oversight	Ethics Committee at the Medical Faculties of the University Heidelberg

Note that full information on the approval of the study protocol must also be provided in the manuscript.

Field-specific reporting

Please select the one below that is the best fit for your research. If you are not sure, read the appropriate sections before making your selection.

[x] Life sciences [ ] Behavioural & social sciences [ ] Ecological, evolutionary & environmental sciences

For a reference copy of the document with all sections, see nature.com/documents/nr-reporting-summary-flat.pdf

Life sciences study design

All studies must disclose on these points even when the disclosure is negative.

Sample size	Mouse single-cell RNA-seq:<br>We analyzed four groups (APP23-tg and WT mice at early and late stages; n = 4 mice per group). Group sizes were chosen based on standard practice in single-cell studies, where 3-5 biological replicates provide sufficient power to detect genotype- and stage-dependent transcriptional changes. Each mouse yielded thousands of high-quality cells, ensuring robust statistical comparisons.<br>Mouse Immunofluorescence:

	No statistical methods were used to predetermine sample size. Sample sizes are consistent with those commonly used in the field and allowed us to obtain reproducible results across experiments.<br>Mouse spatial transcriptomics:<br>Spatial RNA profiling using Molecular Cartography™ was performed on cortical sections from n = 2 mice per group (8 total). The sample size reflects the high data density and technical complexity of spatial transcriptomics, where 1-2 biological replicates per condition are commonly used to capture reproducible spatial expression patterns.<br>Human spatial transcriptomics:<br>Spatial transcriptomics was performed on cortical tissue from n = 4 Alzheimer's disease patients, with two sections per patient analyzed. Sample size was determined by availability of high-quality post-mortem tissue and is consistent with typical human spatial transcriptomic studies, while allowing assessment of inter-individual variability.
Data exclusions	No data-exclusions of assayesd samples were made
Replication	Immunohistochemical analyses were performed in batches of varying numbers and the cumulative data presented in the manuscript. the total n of animals for each analysis has been provided in the corresponding figure legends in the manuscript. Single-cell RNA and VDJ sequencing and targeted single cell spatial transcriptomics were performed once with biological replicates in accordance with current standards.
Randomization	Experimental groups were genotype and age-dependent so no randomization was performed.
Blinding	Investigators were not blinded to group allocation during data collection and analysis. Immunohistochemcial stainings,image acquisition and image processing was performed in a blinded manner. Blinding was not feasible for computational analyses, as sample identities were encoded in the metadata and required for downstream processing. For experimental assays, objective, standardized quantification methods were used to minimize bias.

Reporting for specific materials, systems and methods

We require information from authors about some types of materials, experimental systems and methods used in many studies. Here, indicate whether each material, system or method listed is relevant to your study. If you are not sure if a list item applies to your research, read the appropriate section before selecting a response.

Materials & experimental systems

n/a	Involved in the study
☐	☒ Antibodies
☒	☐ Eukaryotic cell lines
☒	☐ Palaeontology and archaeology
☐	☒ Animals and other organisms
☒	☐ Clinical data
☒	☐ Dual use research of concern
☒	☐ Plants

Methods

n/a	Involved in the study
☒	☐ ChIP-seq
☐	☒ Flow cytometry
☒	☐ MRI-based neuroimaging

Antibodies

Antibodies used	Flow cytometry antibodies<br>anti-mouse CD45 - BV510 (Clone: 30-F11) 103138 Biolegend<br>anti-mouse CD3 - APC (Clone- 17A2) 100236 Biolegend<br>anti-mouse CD11b - FITC (Clone - M1/70) 101206 Biolegend<br>anti-mouse CD3 - PerCP/Cy5.5 (Clone:17A2) 100218 Biolegend<br>anti-mouse CD8a - Pe/Cy7 (Clone: 53-6.7) 100722 Biolegend<br>anti-mouse CD183 (CXCR3) - APC (Clone: CXCR3-173) 126512 Biolegend<br>TotalSeq C Hashstag antibodies<br>TotalSeq™-C0301 anti-mouse Hashtag 1 Antibody 155861 Biolegend<br>TotalSeq™-C0302 anti-mouse Hashtag 2 Antibody 155863 Biolegend<br>Immunofluorescence antibodies<br>Rabbit anti-mouse/human CD3 (polyclonal) A0452 DAKO (Agilent)<br>Rabbit anti-mouse/human CD31 (polyclonal) ab2836 Abcam<br>Anti-β-Amyloid, 1-16 Antibody (Clone: 6E10) 803014 Biolegend<br>Goat anti-mouse IgG (H+L) Secondary antibody AF488 (Polyclonal) A-11029 Invitrogen (Thermo Fisher Scientific)<br>Goat anti-rabbit IgG (H+L) Secondary antibody AF546 (Polyclonal) A-11010 Invitrogen (Thermo Fisher Scientific)<br>Goat anti-rabbit IgG (H+L) Secondary antibody AF633 (Polyclonal) A-21070 Invitrogen (Thermo Fisher Scientific)
Validation	Antibodies were used at concentrations recommended by the suppliers. No additional in-house validation was performed for applications other than histology, and validation information provided by the manufacturers is available on their website (See Methods for catalogue numbers). or histological applications, antibodies were titrated to establish optimal staining conditions; final dilutions and incubation parameters are detailed in the Methods section

Animals and other research organisms

Policy information about studies involving animals; ARRIVE guidelines recommended for reporting animal research, and Sex and Gender in Research

Laboratory animals	APP23-tg mice, full name - Heterozygote B6, D2-TgN[Thy-APPSWE]-23- tg mice, contain a human amyloid precursor protein (APP751) cDNA with the Swedish double mutation at position 670/671 under the control of the neuron-specific Thy-1 promoter and were obtained from Novartis Institutes for BioMedical Research, Novartis Pharma AG, Basel, Switzerland.<br>APP23-tg mice were backcrossed twice with C57BL/6 mice (obtained from Janvier, Saint Berthevin Cedex, France).<br>Wild-type (WT) littermates were used as control animals<br>The Gt(ROSA)26Sor^tm1.1(CAG-cas9*,-EGFP) Fezh mouse line was aquired from Jackson Laboratory. It carries a Cas9-EGFP construct inserted into the Rosa26 locus, allowing ubiquitous expression of Cas9 and EGFP under the control of the CAG promoter. Wildtype (Cas9-WT) littermates were used as donor animals for T-cell isolation in corresponding experiments.
Wild animals	N/A
Reporting on sex	Both male and female mice were included in the study. Sex-dependent differences were assessed for amyloid plaque burden and behavioral outcomes (nestlet assay), where female APP23-tg mice exhibited higher parenchymal plaque burden and lower nestlet-shredding scores compared to males. For downstream single-cell and spatial transcriptomic analyses, animals of both sexes were included within each experimental group. The study was not powered to detect sex-specific differences in immune phenotypes, and sex was therefore not used as a stratification variable in transcriptomic analyses.
Field-collected samples	N/A
Ethics oversight	All animal procedures followed the institutional laboratory animal research guidelines and were approved by the relevant governmental authorities (Regional Administrative Authority Karlsruhe, Germany)

Note that full information on the approval of the study protocol must also be provided in the manuscript.

Plants

Seed stocks	N/A
Novel plant genotypes	N/A
Authentication	N/A

Flow Cytometry

Plots

Confirm that:

- [x] The axis labels state the marker and fluorochrome used (e.g. CD4-FITC).
- [x] The axis scales are clearly visible. Include numbers along axes only for bottom left plot of group (a 'group' is an analysis of identical markers).
- [x] All plots are contour plots with outliers or pseudocolor plots.
- [x] A numerical value for number of cells or percentage (with statistics) is provided.

Methodology

Sample preparation	Mentioned in detail in Methods section of the Manuscript
Instrument	Fluorescene activated cell sorting was performed on BD FACSAriaII (BD Biosciences). Analytic flow cytometry was performed on ZE5 Cell analyzer (Biorad)
Software	Data acquisition for Fluorescene activated cell sorting was done using the BDFACSDiva Software (BD Bioseinces). Data acqusition for Flow cytometry was done using the integrated Everest Software (Biorad)<br>All analysis of flow cytometry data was done on FlowJo v10.
Cell population abundance	Provided in detail in Manuscript figures and supplementary information

Gating strategy	Gating strategy for flow cytometry based cell sorting for single cell RNA and VDJ seq has been shown in Fig S2.<br>Gating strategy for flow cytometric analyis for transwell migration assays has been show in Fig S9

☒ Tick this box to confirm that a figure exemplifying the gating strategy is provided in the Supplementary Information.
